# High-Resolution Peripheral Quantitative Computed Tomography (HR-pQCT) for Assessment of Avascular Necrosis of the Lunate

**DOI:** 10.3390/jimaging11110406

**Published:** 2025-11-12

**Authors:** Esin Rothenfluh, Georg F. Erbach, Léna G. Dietrich, Laura De Pellegrin, Daniela A. Frauchiger, Rainer J. Egli

**Affiliations:** 1 Department of Plastic and Hand Surgery, Inselspital, Bern University Hospital, 3010 Bern, Switzerland; lena.dietrich@insel.ch (L.G.D.); laura.depellegrin@insel.ch (L.D.P.); 2 Department of Orthopaedic Surgery and Traumatology, Inselspital, Bern University Hospital, Medical Faculty, University of Bern, 3010 Bern, Switzerland; georg.erbach@insel.ch; 3 ARTORG Center for Biomedical Engineering Research, University of Bern, 3010 Bern, Switzerland; daniela.frauchiger@unibe.ch; 4 Department of Diagnostic, Interventional and Pediatric Radiology, Inselspital, Bern University Hospital, University of Bern, 3010 Bern, Switzerland; rainer.egli@insel.ch

**Keywords:** avascular necrosis, AVN, Kienböck, lunate, HR-pQCT, quantitative computed tomography

## Abstract

This exploratory study investigates the feasibility and diagnostic value of high-resolution peripheral quantitative computed tomography (HR-pQCT) in detecting structural and microarchitectural changes in lunate avascular necrosis (AVN), or Kienböck’s disease. Five adult patients with unilateral AVN underwent either MRI or CT, alongside HR-pQCT of both wrists. Imaging features such as subchondral remodeling, joint space narrowing, and bone fragmentation were assessed across modalities. HR-pQCT detected at least one additional pathological feature not seen on MRI or CT in four of five patients and revealed early subchondral changes in two contralateral asymptomatic wrists. Quantitative measurements of bone volume fraction (BV/TV) further indicated altered trabecular structure correlating with disease stage. These findings suggest that HR-pQCT may offer enhanced sensitivity for early-stage AVN and better delineation of disease extent, which is critical for informed surgical planning. While limited by small sample size, this study provides preliminary evidence supporting HR-pQCT as a complementary imaging tool in the assessment of lunate AVN, with potential to improve early detection, staging accuracy, and individualized treatment strategies.

## 1. Introduction

The pathophysiological mechanisms of avascular necrosis (AVN) of the lunate, also known as Morbus Kienböck, remain incompletely understood, but the expected course of disease progression is more clearly delineated in the literature [1,2,3,4,5]. Multiple etiological factors have been proposed, including vascular insufficiency, repetitive microtrauma, negative ulnar variance, and biomechanical alterations within the carpal architecture. Despite extensive research, the interplay between these contributing factors and the sequence of histopathological changes leading to subchondral collapse is still debated. It is generally accepted that compromised vascular supply to the lunate plays a central role in the onset of ischemic necrosis, which subsequently leads to trabecular bone resorption, structural weakening, and eventual collapse. The lunate’s unique vascular anatomy, characterized by variable intraosseous anastomoses and dependence on small dorsal and volar branches of the radial and ulnar arteries, renders it particularly vulnerable to circulatory disturbances. Mechanical overload, either due to ulnar variance or altered carpal kinematics, may exacerbate ischemic injury by increasing intraosseous pressure and impairing revascularization Early-stage diagnosis may facilitate timely intervention, which in turn could potentially decelerate disease progression [6]. Magnetic resonance imaging (MRI) has proven valuable in detecting AVN of the lunate at an early stage, when clinical symptoms may already be present despite unremarkable radiographic findings [7]. MRI also assists in excluding differential diagnoses, such as ganglion cysts or trauma-related edema, which resolve over time. For staging AVN of the lunate, however, computed tomography (CT) is generally preferred, as it offers superior visualization of structural changes such as fractures, bone fragmentation, and osseous integrity [8,9]. High-resolution peripheral quantitative computed tomography (HR-pQCT), a low-dose, high-resolution three-dimensional imaging modality, further enhances imaging capabilities by providing detailed visualization of bone microarchitecture [10,11,12,13,14]. The enhanced resolution of HR-pQCT enables the detection of early microstructural deterioration and morphological changes in the lunate and adjacent carpal bones, potentially improving the accuracy of staging. Importantly, HR-pQCT also offers a quantitative evaluation of bone parameters—such as trabecular density, cortical thickness, and volumetric bone mineral density (vBMD)—which may provide insights into the mechanical integrity of the affected bone [15,16,17]. Such information could help predict structural failure and guide the choice between joint-preserving and salvage surgical procedures.

A further advantage of HR-pQCT lies in its potential preoperative utility: by delineating the extent of articular surface involvement and assessing subchondral bone integrity, it may enable more precise surgical planning and tailored interventions. In contrast, conventional imaging modalities, including standard radiographs, routine CT, and MRI, may underestimate the degree of joint compromise, possibly leading to delayed or suboptimal treatment decisions, such as the timing of proximal row carpectomy or partial arthrodesis.

In this study, we compare the imaging characteristics of MRI and standard-resolution CT with those of HR-pQCT in the evaluation of lunate AVN, focusing on the visualization of the articular surfaces and assessment of overall osseous integrity. Furthermore, we analyze quantitative HR-pQCT parameters of the lunate’s bone microarchitecture in comparison with the contralateral, unaffected side. Particular attention is given to differences in trabecular bone mineral density, with the aim of refining risk assessment and identifying potential imaging markers of disease severity and surgical outcomes for future clinical application.

## 2. Materials and Methods

### 2.1. Aim, Design and Setting

The aim of this exploratory study was to assess the feasibility and diagnostic potential of HR-pQCT for evaluating lunate AVN. The study was designed as a cross-sectional observational study conducted at a University Hospital, with a dedicated Hand Surgery Department and a Department for Diagnostic, Interventional and Pediatric Radiology with specific expertise in musculoskeletal imaging.

### 2.2. Participants and Materials

Five adult patients with clinically and radiologically confirmed unilateral lunate AVN were included. Patients with prior wrist surgery or systemic bone disorders were excluded. Imaging findings were assessed using MRI in three patients (patients 1–3) and standard resolution CT in two patients (patients 4–5). In addition, all patients underwent HR-pQCT imaging of both the affected and contralateral (healthy) wrists within six weeks of the other imaging modality in four patients, and within ten weeks in one patient.

### 2.3. Processes, Interventions and Comparisons

HR-pQCT was performed with the 2nd Generation of the XtremeCT (Scanco Medical, Brüttisellen, Switzerland). For the examination, the X-ray tube was operated at 68 kVp and 1.47 mA. The image matrix size was set to 1024 × 1024, with a nominal isotropic voxel size of 61 μm, for each of the three required stacks. One stack covers a height of 10.2 mm and consists of 168 slices. The reference lines for the two stacks were manually aligned to encompass the region of interest in the carpus. Using a double-stack acquisition offers the following advantages: (1) the region of interest available for longitudinal studies is doubled, (2) a patient size-dependent region of interest can be defined during post-processing rather than during the clinical examination.

Imaging features associated with lunate AVN were assessed using MRI, CT and HR-pQCT. Evaluated criteria included:

- Fracture displacement (>2 mm)
- Lunocapitate and radiolunate joint space narrowing
- Subchondral bone remodeling
- Internal bone fragmentation
- Surface bone fragmentation

These features were assessed on MRI and CT of the affected side by a blinded expert musculoskeletal radiologist. The same criteria were then evaluated on HR-pQCT images of the affected side to determine the modality’s capability. All image analyses, including HR-pQCT evaluations, were performed by a blinded expert musculoskeletal radiologist who was unaware of the clinical diagnosis and of the findings from other imaging modalities. HR-pQCT scans were acquired for both the affected and contralateral sides, enabling intra-individual comparison.

**Figure 1:**
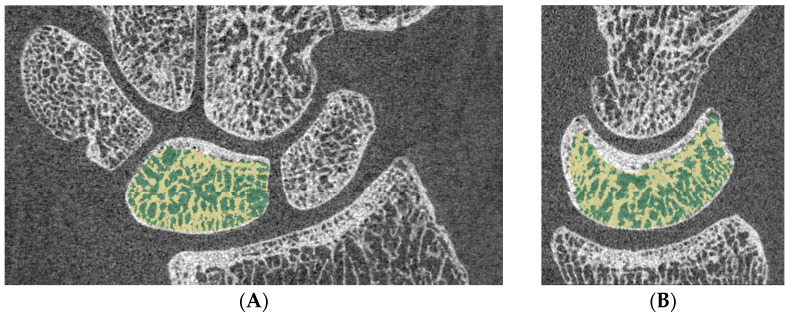
(**A**) Segmentation of the lunate bone in coronal view (green = total volume, yellow = bone volume) (**B**) sagittal view.

**Figure 2:**
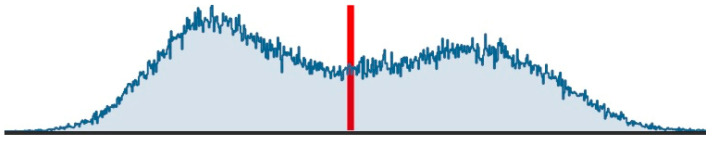
The attenuation histogram. Red indicator denotes 1400 relative HU (“valley”, global threshold confirmed visually for segmentation accuracy). Range −1000 to 3500 relative HU. Peak 1 (background) at 250, and peak 2 at 2500 relative HU.

Additionally, HR-pQCT was used to assess trabecular structure and density. All segmentation procedures were performed in 3D Slicer (version 5.6.2; RRID:SCR_005619) (Figure 1) [18]. Cortical and trabecular components were separated using manual contouring, which also defined the Total Volume (TV) for each sample. Bone Volume (BV) was segmented by applying a global threshold derived from the attenuation histogram, selecting the valley (i.e., minimum point) between background and bone peaks [19] (Figure 2). HR-pQCT grey-level values represent scanner-specific relative attenuation units. A global threshold of 1400 relative HU was applied consistently across samples, corresponding to the histogram valley between soft tissue and mineralized bone. Segmentation quality was visually inspected for each dataset to confirm accurate inclusion of trabecular bone and exclusion of background, in accordance with Bouxsein/JBMR guidelines [15,20,21]. Minor threshold variations did not affect bone delineation, confirming robustness of the chosen value and aligning with previous literature [22,23]. The chosen threshold corresponded to a valley within the range of −1000 to 3500 relative HU, between a background peak at approximately 250 HU and a bone peak at approximately 2500 HU, with a cutoff of 1400 relative HU used to define the bone volume fraction. Trabecular density was characterized by calculating the BV/TV ratio and comparing it with the contralateral side.

### 2.4. Data Interpretation

Due to the small sample size (*n* = 5), no formal statistical analysis was performed. Instead, findings were described qualitatively, and trends were noted across imaging modalities. This pilot approach was intended to inform the design of future larger-scale studies.

## 3. Results

Of the five patients, three were male and two were female. The average age of the five patients was 36 years (range 21–44 years).

### 3.1. MRI Findings (Patients 1–3)

Two out of three demonstrated displacement greater than 2 mm. Subchondral remodeling was observed in all MRI cases. Lunocapitate (LC) joint space narrowing was present in two of the three, while no patients showed radiolunate (LR) joint space narrowing. Internal fragmentation was detected in one instance, and surface fragmentation in another.

### 3.2. CT Findings (Patients 4–5)

Both CT scans showed fracture displacement > 2 mm and subchondral remodeling. LC joint space narrowing was present in one case, while the other patient showed narrowing of the LR joint space. Surface fragmentation was observed in both CT scans, but internal fragmentation was not seen. Table 1 is a summary of all findings based on the conventional imaging modalities.

### 3.3. HR-pQCT Against Standard Imaging Modalities (MRI and CT)

Results for HR-pQCT were compared against standard imaging (MRI and CT) on the affected side for each patient and are summarized in Table 2. In four of the five patients, at least one criterion was identified with HR-pQCT but not with the other imaging techniques. These discrepancies most commonly involved LC or LR joint narrowing, subchondral remodeling, and surface fragmentation, suggesting that HR-pQCT may detect alterations in surface morphology and integrity at a lower threshold for pathological change.

### 3.4. HR-pQCT Results: Affected Versus Non-Affected Sides

Results from HR-pQCT of the affected side (a) are compared with the contralateral, non-affected side for each patient and summarized in Table 3. In two patients, subchondral remodeling was also observed on the non-affected side, suggesting a potential early indicator of AVN. As no conventional imaging was performed for the contralateral side, a direct comparison of HR-pQCT with standard MRI or CT for early detection could not be undertaken.

### 3.5. Density and Structure Measurement in the HR-pQCT

**Figure 3:**
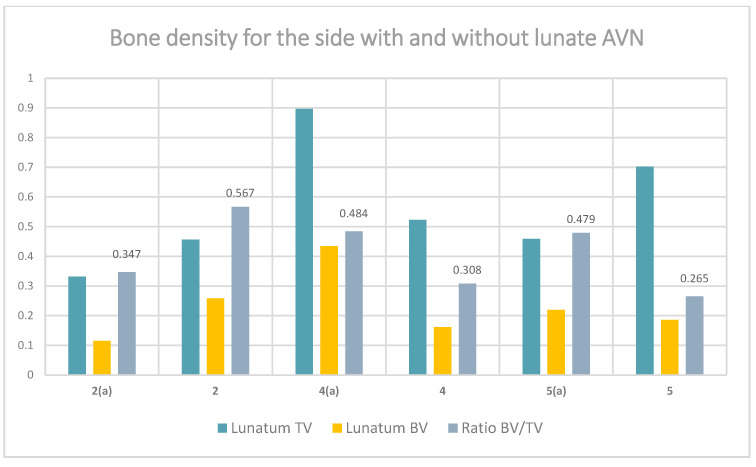
Results of bone density structural measurement by HR-pQCT for the pathological (a) and contralateral sides, expressed as Lunatum TV and BV and the BV/TV ratio (BV = bone volume; TV = total volume). The numbers on the horizontal axis stand for the patient Index. Patient 2 suffers from an early stage of AVN, patients 4 and 5 suffer from late stages of AVN.

Results for the total lunate volume and the bone volume are summarized in Table 4 and Figure 3. The ratio is increased on the affected side in cases of higher degrees of AVN of the lunate (>IIIa according to the Lichtmann classification). For patients 1 and 3, no sufficient segmentation could be performed due to advanced collapse, fragmentation and resorption within the lunate bone. For these two cases, only the contralateral side was evaluated. For patients 2, 4 and 5 a paired t-test was run, yielding a mean difference of 0.0567 (affected—contralateral), with a 95% confidence interval of −0.5404 to 0.6537 and a *p*-value of 0.723 (α = 0.05). Given the very small sample size (*n* = 3) the *p*-value should not be interpreted as a robust statistical indicator.

### 3.6. Clinical Outcome and Follow-Up

All five patients underwent HR-pQCT scanning prior to any salvage procedure. Patients 1–3 subsequently received a proximal row carpectomy (PRC) and resurfacing of the capitate using a pyrocarbon implant (RCPI). Among them, Patient 1 was very satisfied with the outcome and had the final follow-up at 16 months postoperatively. Patient 2 underwent conversion to a total wrist arthrodesis three years after PRC and RCPI due to refractory pain and progression to a stage IV AVN. Patient 3 continued to experience pain up to the final follow-up 32 months postoperatively and was lost to further follow-up. Patient 4 had previously undergone a metaphyseal decompression 22 years earlier and presented for consultation following a new injury to the same wrist. As the AVN had progressed to stage IIIa, a PRC and RPNI procedure was recommended. However, after HR-pQCT scanning, the patient did not return for surgery and was lost to follow-up. Patient 5 was advised to undergo a salvage procedure but declined and did not return to the clinic.

## 4. Discussion

HR-pQCT was originally developed for osteoporosis assessment and microstructural evaluation of the distal tibia and radius. Since the introduction of the first generation (XtremeCT/XCT) in 2004 and its successor (XtremeCT II/XCT2) in 2014, which offers improved resolution (61 versus 82 μm) and reduced scanning time, the potential applications of HR-pQCT have expanded considerably. A major strength of HR-pQCT is its applicability in instances where subtle changes are anticipated, such as during the progression within Lichtman stage III [9,24].Despite these advances, routine clinical integration outside research settings has not yet been achieved. Barriers include susceptibility to motion artifacts, operator- dependent variability, limited cost-effectiveness data, and the absence of standardized imaging protocols. Furthermore, existing normative datasets remain insufficient in size and diversity, underscoring the need for larger, cross-validated cohorts to establish clinical feasibility.

We propose a novel diagnostic application of HR-pQCT for AVN of the lunate. Our findings suggest that HR-pQCT can provide additional insights into disease detection and staging compared with conventional CT or MRI. Importantly, the dataset includes both pathological and contralateral healthy wrists, allowing within-patient comparisons. Given that AVN of the lunate bone may be bilaterally present [25], contralateral imaging offers an opportunity to detect early microstructural changes. Indeed, in two of the five patients, HR-pQCT identified subchondral remodeling in the clinically asymptomatic contralateral lunate, potentially serving as an early indicator of AVN. The Bone volume fraction (BV/TV) varied with AVN severity in our series, showing increased volumetric density in advanced (≥stage III) AVN, consistent with sclerosis, and reduced density in earlier stages. HR-pQCT also enabled recognition of staging-relevant features such as subchondral remodeling, surface fragmentation, and intercarpal joint narrowing, some of which were not evident on CT or MRI. This information may be highly relevant for surgical planning, where the choice between joint-preserving and salvage procedures relies heavily on accurate staging. Taken together, our observations provide preliminary evidence that HR-pQCT may complement existing imaging modalities in the assessment of lunate AVN. The consistent detection of subchondral remodeling and surface fragmentation suggests these may represent useful HR-pQCT targets for disease evaluation. It should be noted that HR-pQCT was not employed for surgical planning in this exploratory study. The examinations were performed solely for research purposes to assess the modality’s potential value for future clinical application. Looking forward, quantitative parameters derived from HR-pQCT—such as bone stiffness and load-to-failure estimates—could be incorporated into finite element analysis (FEA) models, thereby enhancing biomechanical characterization of the lunate [26]. This integration could offer new opportunities to refine staging, predict fracture risk, and inform personalized surgical strategies.

Lunate AVN (Kienböck’s disease) remains challenging to diagnose in its early stages, as radiographic findings often lag behind symptoms, and MRI—while sensitive—does not always allow precise staging. Authors such as Stahl et al. emphasized that while MRI is sensitive to early marrow signal alterations, it provides insufficient spatial resolution to assess subchondral or cortical integrity, leading to diagnostic uncertainty particularly in intermediate and late stages [9,27]. Microstructural assessment with HR-pQCT could address this gap by providing quantitative parameters such as trabecular thickness, number, and separation, which may reflect disease progression earlier than conventional methods. In the context of surgical decision-making, the identification of surface irregularities, subchondral remodeling, and intercarpal joint narrowing by HR-pQCT is noteworthy. These features are critical when considering procedures such as revascularization, osteotomy, or salvage arthrodesis, where accurate evaluation of lunate integrity determines treatment success. HR-pQCT imaging results could have a relevant impact in decision-making to uptake more aggressive treatment strategies and prevent further progression of joint damage. Our findings therefore align with the growing recognition that high-resolution imaging modalities can advance personalized management strategies in AVN. Developing tools to enable earlier diagnosis would be highly beneficial for lunate AVN, and accurate deep-learning (DL) models could serve as valuable screening and staging aids [28]. In particular, convolutional neural networks (CNNs) trained on HR-pQCT data may achieve human-level or superior accuracy in detecting and staging the disease.

A focused literature search revealed very few in vivo HR-pQCT studies directly addressing the lunate or Kienböck’s disease. Most recent publications (2024–2025) address HR-pQCT methodology, segmentation, or applications in small peripheral bones more broadly rather than lunate-specific clinical series [9,29,30]. This scarcity of direct evidence illustrates the gap that our exploratory cohort aims to begin to address; consequently, our findings are presented as preliminary, hypothesis-generating observations to inform future, larger studies.

This exploratory study has several limitations that should be considered when interpreting the results. Foremost, the small sample size of five patients limits any generalizability, and no formal statistical analysis could be performed, because the study was not powered for inferential testing. Instead, the analysis focused on descriptive and qualitative assessment to identify feasibility. The observations should therefore be regarded as preliminary and hypothesis-generating, intended to inform and guide future, larger-scale studies. Furthermore, not all participants underwent the same reference imaging modality, as three received MRI and the others CT. This heterogeneity may have introduced variability in detecting specific pathological features, particularly give the differing sensitivities of MRI for marrow changes and CT for cortical or subchondral integrity. While the comparison modalities differ, each provided complementary structural information relevant to evaluating HR-pQCT’s potential clinical utility. The lack of histopathological confirmation also prevents definitive validation of HR-pQCT findings against disease progression or tissue-level changes. Future studies with larger and more homogeneous cohorts and longitudinal assessment are needed to establish the clinical utility of HR-pQCT in Kienböck’s disease.

## 5. Conclusions

Our findings suggest that HR-pQCT has the potential to complement conventional imaging in the early detection and staging of lunate AVN by capturing microstructural and surface changes not visible with CT or MRI. While preliminary, these results highlight its promise for refining surgical decision-making and support further validation in larger patient cohorts.

## Figures and Tables

**Table 1:** Summary of criteria fulfilled on conventional imaging, reported as the number of cases (n) and the percentage of the total patient cohort. Surface fragmentation was observed in all patients, all of whom also demonstrated fracture displacement greater than 2 mm.

Assessment Criteria on Standard Imaging		Number of Patients (Percentage, %)
Fracture displacement > 2 mm		4 (80%)
Subchondral remodeling		5 (100%)
Internal fragmentation		1 (20%)
Surface fragmentation		3 (60%), all with fracture displacement > 2 mm
Articular space narrowing	LC joint	3 (60%)
	LR joint	1 (20%)

**Table 2:** Results of HR-pQCT compared with standard imaging for each patient. Shaded cells indicate criteria detected on HR-pQCT but not visible on standard imaging.

Patient	Imaging Modality	Fracture Displacement	LC Joint Narrowing	LR Joint Narrowing	Subchondral Remodeling	Internal Fragmentation	Surface Fragmentation
1	MRI	>2 mm	Yes	No	Yes	Yes	Yes
	HR-pQCT	>2 mm	Yes	Yes	Yes	Yes	Yes
2	MRI	<2 mm	No	No	Yes	No	No
	HR-pQCT	<2 mm	No	No	Yes	No	Yes
3	MRI	>2 mm	Yes	No	Yes	No	No
	HR-pQCT	>2 mm	Yes	No	Yes	No	No
4	CT	>2 mm	No	No	No	No	Yes
	HR-pQCT	>2 mm	Yes	No	Yes	No	Yes
5	CT	>2 mm	No	No	Yes	No	Yes
	HR-pQCT	>2 mm	No	Yes	Yes	No	Yes

**Table 3:** HR-pQCT results are compared between the pathological and the contralateral sides. Shaded cells indicate cases, in which one or more criteria were also detected on the contralateral side. Letter (a) indicates the affected side.

Patient	Imaging Modality	Fracture Displacement	LC Joint Narrowing	LR Joint Narrowing	Subchondral Remodeling	Internal Fragmentation	Surface Fragmentation
1 (a)	HR-pQCT	>2 mm	Yes	Yes	Yes	Yes	Yes
1	HR-pQCT	No	No	No	No	No	No
2 (a)	HR-pQCT	<2 mm	No	No	Yes	No	Yes
2	HR-pQCT	No	No	No	Yes	No	No
3 (a)	HR-pQCT	>2 mm	Yes	No	Yes	No	No
3	HR-pQCT	No	No	No	Yes	No	No
4 (a)	HR-pQCT	>2 mm	No	No	No	No	Yes
4	HR-pQCT	No	No	No	No	No	No
5 (a)	HR-pQCT	>2 mm	No	No	Yes	No	Yes
5	HR-pQCT	No	No	No	No	No	No

**Table 4:** Results of bone density structural measurement by HR-pQCT for the pathological and contralateral sides, expressed as the BV/TV ratio (BV = bone volume in cm^3^; TV = total volume in cm^3^). Letter (a) denotes the affected side. Light-shaded cells represent the cases of advanced AVN, where the ratio is elevated on the affected side due to sclerotic changes. Dark-shaded cells represent a case in which the affected side shows a reduced ratio, corresponding to an earlier stage of AVN (stage IIb according to Lichtmann). Voxel size = 61 µm (isotropic). Voxel volume = 61^3^ = 226,981 µm^3^ = 2.27 × 10^−7^ cm^3^. BV and TV were calculated as voxel count × voxel volume.

Patient	Lunatum TV (cm^3^)	Lunatum BV (cm^3^)	Ratio BV/TV
1 (a)			
1	1.03639	0.341963	0.330
2 (a)	0.33167	0.115174	0.347
2	0.456183	0.258456	0.567
3 (a)			
3	1.39478	0.563206	0.404
4 (a)	0.896641	0.434144	0.484
4	0.523274	0.161169	0.308
5 (a)	0.458871	0.219874	0.479
5	0.70271	0.186203	0.265

## Data Availability

The data presented in this study are available on request from the corresponding author due to protect privacy.

## Abbreviations

The following abbreviations are used in this manuscript:

HR-pQCT	High resolution peripheral quantitative computed tomography
AVN	Avascular necrosis
LC	Lunocapitate
LR	Radiolunate
